# Researches Concerning Compensatory Diarrhea

**Published:** 1908-08

**Authors:** 


### CORRESPONDENCE

### Researches Concerning Compensatory Diarrhea.

**Dr. George N. Jack Discusses a Paper with this Title Read at the Chicago Meeting of the A. M. A., by Dr. Heinrich Stern.**

*Editor Buffalo Medical Journal.*

Sir: As other medical journals are publishing discussions and abstracts of papers that were read at the A. M. A. meeting, I thought it would not be out of place for you to publish my discussion of Dr. H. Stern's paper, since your Journal has published so many of my papers, your readers naturally would be interested in this discussion.

I enclose discussion for your kind consideration.

G. N. JACK.

Buffalo, June 17, 1908.

The Chicago A. M. A. meeting brought out its usual grist of papers and discussions, all of which lend their mite toward the solution of the unsolved problems of life.

Some very interesting discussions were brought out, not the least of which was Dr. George N. Jack's discussion of Dr. Heinrich Stern's paper on "Researches Concerning Compensatory diarrhea."

DISCUSSION.

Dr. Jack, Buffalo.—I was much interested in Dr. Stern's paper. I have, as you all have, often seen eczema, sick headache and certain forms of asthma alternate with diarrhea. Now why is this? I, while pursuing my investigation along a certain special line have tumbled, as a result of operations, experiments on dogs, cadavers, etc., upon a few physiological respiratory laws that, while they are not in accord with authoritatively established opinions, they, to my mind, very satisfactorily explain the relationship between certain asthmas and a compensatory diarrhea, and if you wish I will give them to you:

1. Lung tissue has a glandular structure, and performs a glandular function, which therefore causes the lungs to actually constitute two large irregular lymphangio-pneumatic glands. In fact lung tissue is of a glandular nature and function serving the mortar like purpose of holding the air tubes, bloodvessels and lymphatics in close or functionating approximation.

2. The elasticity of the lungs is not so pronounced as it has been believed to be, neither does it constitute the expiratory force given it by existing authorities.

3. Lung tissue of itself, not including the elastic visceral pleura or capsule covering it, nor the numerous small bloodvessels, everywhere ramifying it, is a coarse, friable, granular non-elastic glandular like tissue of little resistance that easily pick to pieces, tears or pulls apart in irregular, rough, ragged chunks of all sizes and shapes. Lung tissue by itself, therefore is not elastic as it appears en masse.

4. The only elastic tissues that acts directly upon the lung substance is that of the visceral pleura, and the numerous small bloodvessels or capillaries that so abundantly ramify the lung.

5. The lung tissue itself is practically *passive* during both inspiration and expiration.

6. Owing to the vacuum like pull between the visceral and parietal pleuræ, the lungs are as it were glued to the thoracic walls, from which they cannot pull, and they are passively expanded and passively compressed by the action of these walls.

7. The lungs collapse much more readily and completely on puncturing the thoracic wall of the cadaver, than from the same proceedure in the living subject owing to the *lack* of pulmonary or lung blood pressure in the cadaver.

8. The existence of the pulmonary or lung blood pressure in the living subject tends to render the lungs more or less erotic thus serving to prevent them from collapsing so readily and completely in the living subject as in the cadaver upon admitting atmospheric air between the visceral and parietal pleurae.

9. The air cells, minus the numerous little bloodvessels in their walls, are not elastic, but they are very collapsible. The air cells do not contract to force out the inspired air, but they are made to partially collapse and thus caused to shove the inspired air out.

The normal expiratory partial collapse of the air cells in normal expiration is due to the pressure of the thoracic wall upon the air cell, the recoil of the elastic visceral pleura or lung capsule, and air cell wall capillaries, and to the expiratory changes in the pulmonary blood pressure, said blood pressure exerting its influence through the dense capillary net-work of bloodvessels found upon the walls of the air cells.

Thus a high blood pressure retards the collapsibility of the air-cells and favors expiratory dyspnea, which physiologically generally furnishes the last straw in the production of the prolonged expiratory wheeze of the air tube plugged asthmatic, and hence the immediate relief in certain asthmas following nausea, chloroform, the nitrites, altitude, or other agents which tend to reduce the pulmonary blood pressure. And on the other hand the death-like dyspnea in the asthmatic following the high blood pressure of exertion, alimentary intoxication, and the like.

The immediate relief from the dyspnea of certain forms of asthma and emphysema following perforation and entrance of air through the thoracic wall is due largely to its anti-blood aspirating effect and resulting pulmonary blood pressure changes.

Conclusions. The apparent elasticity of lung tissue is due to a blood pressure effect.

The *absence* of blood pressure permitting the lungs of the dead to collapse, from the atmospheric pressure in a manner resembling great elastic tension.

The *presence* of blood pressure in the dense capillary network of bloodvessels found upon and practically constituting, the thin walls of the air cells, is what gives to these coils of bloodvessels or air-cells an elastic effect in the lining functionating lung.

The general normal inspiratory rise of blood pressure tends to render the air-cell wall network coil of bloodvessels erotic, thus producing an opening effect on the air-cell which favors the entrance of air, while the normal expiratory reduction of blood pressure favors a softening of the tubular or vascular air-cell wall and thus favors the expiratory collapse of the bloodvessel walled air-cell.

Lung tissue then performs a glandular function while the air, air tubes, blood and bloodvessels do the respiration.

If these laws prove true, and if they are as I claim, founded upon unrecognised physiological laws that can be demonstrated by anatomical facts and laws of physics, they mean much to the future of medicine.

---

If in removing adenoids the mass is seized below and gradually stripped off from the mucous membrane, it can be more quickly and completely removed.

